# Mutiple keratocystic odontogenic tumors (KCOT) in a patient with Gorlin syndrome: a case report with late presentation and absence of skin manifestations

**DOI:** 10.1186/s13104-016-2166-4

**Published:** 2016-07-22

**Authors:** Atif Ali Hashmi, Muhammad Muzzammil Edhi, Naveen Faridi, Mervyn Hosein, Mehmood Khan

**Affiliations:** Department of Histopathology, Liaquat National Hospital and Medical College, Karachi, Pakistan; Liaquat National Hospital and Medical College, Karachi, Pakistan; Department of Maxillofacial Surgery, Liaquat National Hospital and Medical College, Karachi, Pakistan; Dhaka Medical College, Dhaka, Bangladesh

**Keywords:** Gorlin syndrome, Keratocystic odontogenic tumor, Falx cerebral calcifications

## Abstract

**Background:**

Gorlin syndrome is a rare autosomal dominant syndrome characterized by multiple basal cell carcinomas, keratocystic odontogenic tumors (KOT) and falx cerebral calcifications, which occur due to mutation in PTCH gene.

**Case presentation:**

A 36 year old Asian patient presented with jaw swelling and pain. Radiographic examination revealed six cysts in maxilla and mandible which were excised and histologically were compatable with keratocystic odontogenic tumors. CT scan also revealed falx cerebral calcification which led to the diagnosis of Gorlin syndrome confirmed on genetic testing. There was no evidence of basal cell carcinoma and other manifestations of Gorlin syndrome were absent.

**Conclusions:**

Multiple KCOT are hallmark of Gorlin syndrome and should always leads to its suspicion even in the absence of other manifestations and late presentation. Moreover, keratocystic odontogenic tumors have a particularly higher risk of recurrence and patients with Gorlin syndrome are prone to develop additional keratocystic odontogenic tumors from basal cells of oral epithelium. Therefore we suggest a stepwise approach to manage such patients which include a preoperative biopsy to establish a definitive diagnosis and complete removal of all keratocystic odontogenic tumors to prevent recurrence followed by close clinical follow up and early removal of any newly developed or recurrent cyst. Additionally thorough clinical examination is necessary to rule out the possibility of Gorlin syndrome in any patient with keratocystic odontogenic tumors as there are only subtle differences in histology of those cysts with a syndromic association and clinical features of Gorlin syndrome are markedly variable. Hence late occurrence of keratocystic odontogenic tumors and absence of skin manifestations like basal cell carcinoma should not preclude a diagnosis of Gorlin syndrome.

## Background

Gorlin syndrome is a rare autosomal dominant syndrome characterized by multiple basal cell carcinomas, keratocystic odontogenic tumors (KCOT), rib and vertebral abnormalities, intracranial calcifications, especially of falx cerebri and distinct facial abnormalities [1–3]. Gorlin syndrome was first described in 1960 and its incidence range from 1 in 50,000 to 150,000 [4]. Molecular genetics revealed that Gorlin syndrome is caused by mutation in PTCH gene located on chromosome 9q [5]. Recently, it was also found that hedgehog stimulation and nutritional deprivation synergistically activate the hedgehog signaling pathway in Gorlin syndrome fibroblasts that lead to developmental defects and tumorigenicity [6].

Gorlin/nevoid basal cell carcinoma syndrome (NBCCS) should be suspected in individuals with the following findings [7]:

### Major criteria

- *Lamellar (sheet*-*like) calcification of the falx* or clear evidence of calcification in an individual younger than age 20 years. Falx calcification is nearly always present and is visible on anteroposterior (AP) x-rays of the skull after age 20 years
- *Jaw keratocyst* Odontogenic keratocyst histologically; seen on orthopantogram as an area of translucency
- *Palmar/plantar pits* (two or more); particularly useful in diagnosis and more pronounced when the hands and feet are soaked in warm water for up to 10 min. Pits may appear as white “punched-out” or pink “pin-prick” lesions.
- *Multiple basal cell carcinomas (BCCs)* (>5 in a lifetime) or a BCC before age 30 years. Provision needs to be made for decreased risk of BCC in dark-skinned races and increased risk in whites living in hot sunny climates.
- First-degree relative with NBCCS.

### Minor criteria

- Childhood medulloblastoma (also called primitive neuroectodermal tumor [PNET])
- Lympho-mesenteric or pleural cysts
- Macrocephaly (OFC >97th centile)
- Cleft lip/palate
- Vertebral/rib anomalies observed on chest x-ray and/or spinal x-ray
- Preaxial or postaxial polydactyly
- Ovarian/cardiac fibromas
- Ocular anomalies (cataract, developmental defects, and pigmentary changes of the retinal epithelium).

### Establishing the diagnosis

The diagnosis of NBCCS is established in a proband with the following findings:

- Two major diagnostic criteria and one minor diagnostic criterion or one major and three minor diagnostic criteria.
- Identification of a heterozygous germline *PTCH1* or *SUFU* pathogenic variant on molecular genetic testing. This finding establishes the diagnosis if clinical features are inconclusive.

We describe a rare case of multiple KCOT previously known as odontogenic keratocysts (OKCs) in a patient with Gorlin syndrome.

## Case presentation

**Fig. 1:**
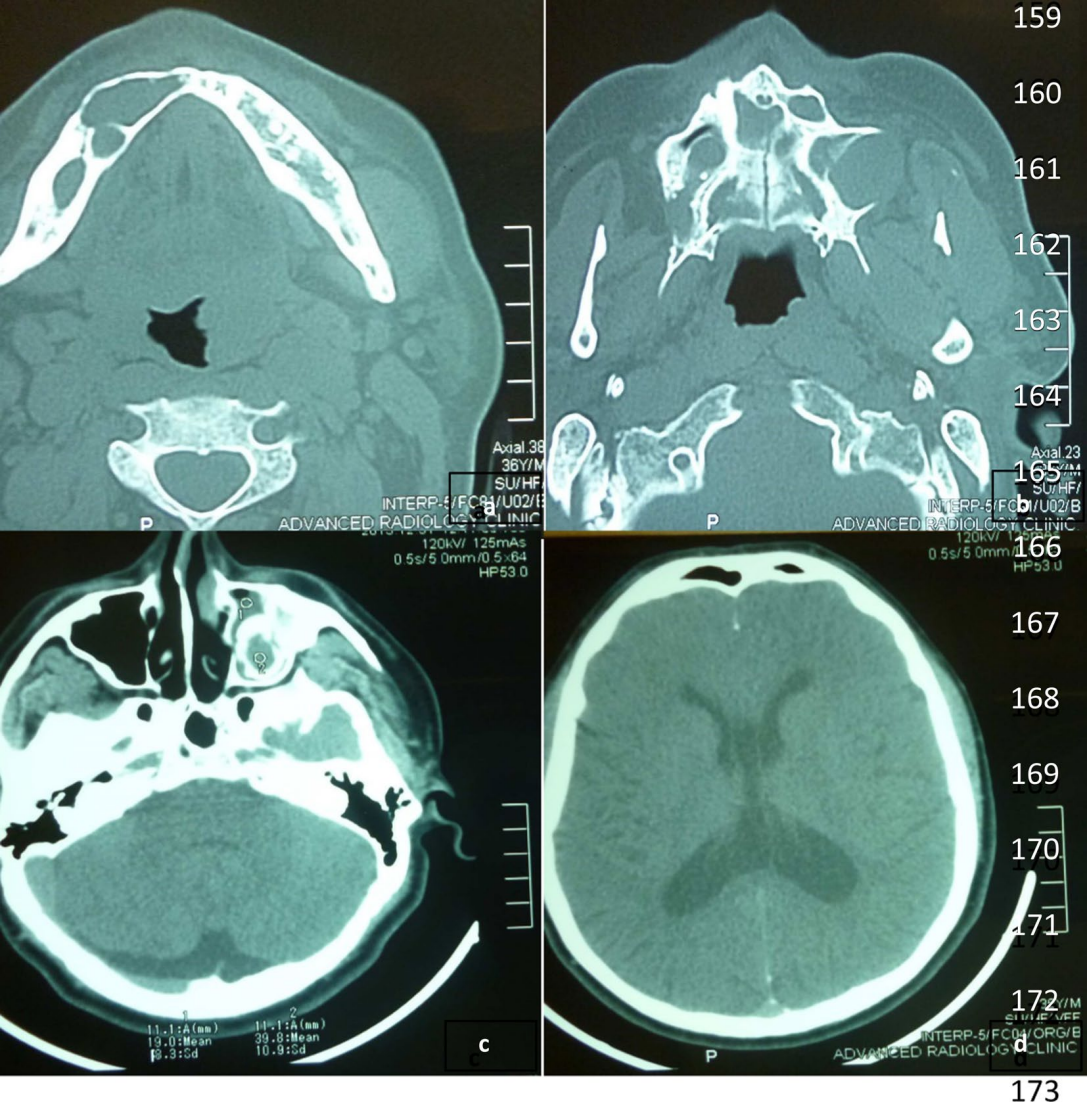
**a**–**c** CT scan showing multiple cystic lesions in maxilla and mandible. **d** CT scan showing falx cerebral calcification

**Fig. 2:**
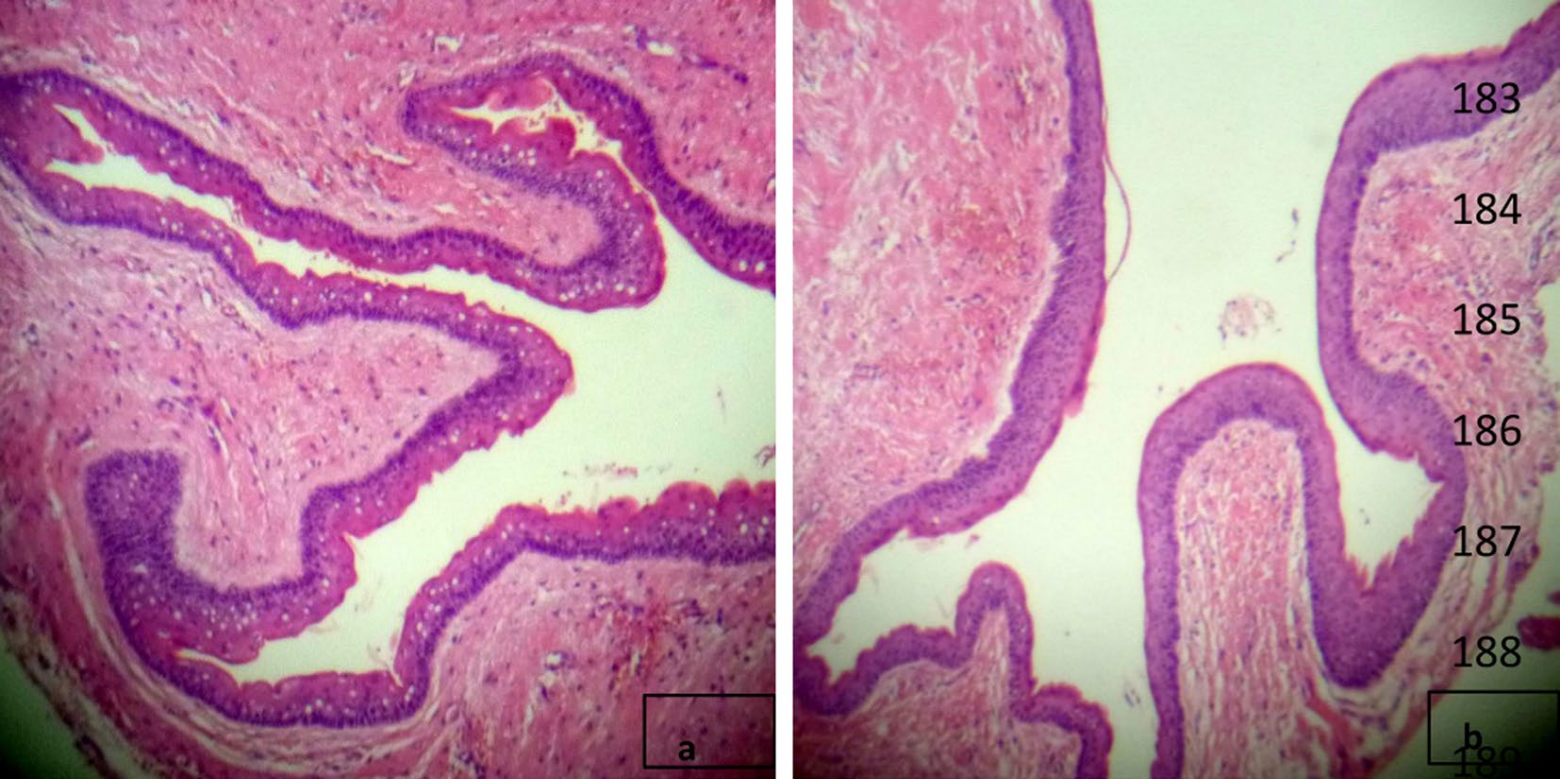
**a**, **b** Histologic sections showing cystic lesions lined by stratified squamous epithelium

A 36 year old Asian patient presented to the dental clinic with complaints of jaw swelling and pain. On intraoral examination there was swelling of both upper and lower jaws with displaced permanent teeth. The swellings were firm in consistency and tender on palpation. Radiographs revealed multiple cysts in both maxilla and mandible. Systemic examination did not reveal any skin lesions. CT scan was performed with revealed three cystic lesions in the maxilla involving right anterior, left anterior and left posterior regions. Three cystic lesions were also observed in the mandible involving right anterior, right posterior and retromolar regions. The largest cyst was found in left posterior maxillary region measuring 3 × 2 cm. Falx cerebral calcifications were also observed (Fig. 1). Excision of all cysts were performed and sent for histopathologic examination. Hematoxylin and eosin sections of all the cysts show lining of stratified squamous epithelium with parakeratosis and corrugated outlines. Areas of calcification along with acute and chronic inflammation in the surrounding tissues were also observed. These histologic findings were consistent with the diagnosis of KCOT (Fig. 2).

The clinical findings of multiple OKCs with falx cerebral calcifications were compatable with the diagnosis of Gorlin syndrome. Moreover, genetic testing revealed PTCH gene mutation. Patient’s family members were screened clinically for the presence of any feature which is suggestive of Gorlin syndrome however; none of them revealed any such finding.

## Discussion

To our knowledge this is the first reported case of Gorlin syndrome in our country. One of the characteristic features of this syndrome is the presence of multiple jaw cysts which may appear in very early life and thus can lead to its recognition at that time. Our case is unique in this aspect that the patient presented in the fourth decade of life and yet there was no skin lesion which can be clinically suspected to be of basal cell carcinoma. One of the explanations may be that the patient is dark skinned not very much involved in outdoor activities which led to his protection from basal cell carcinoma. Another distinctive feature of our case was quite a larger number of cysts compared to most of the case reports where average number of jaw cysts was three. Falx cerebral calcification is another pathognomonic characteristic of Gorlin syndrome which was demonstrated in our case. Saulite et al. reported a case with incidental finding of falx cerebral calcification leading to the diagnosis of Gorlin syndrome [8]. Other characteristic facial features of Gorlin syndrome like cleft lip/palate, micropthalmia were not present in our case.

KCOT occurring in Gorlin syndrome have a higher recurrence rate as compared to nonsyndromic solitary keratocysts [9]. This aggressive behavior may be explained by the presence of daughter and satellite cysts in syndromic keratocysts [10]. In our case daughter cysts were not observed. In addition, two of the cysts were heavily inflamed with focal calcification and giant cell reaction. Biologically, it is believed that in syndromic KCOT epithelial remnants are left behind which give rise to recurrence of cysts and therefore it is suggested that overlying mucosa should be excised before bone grafting [11].

Although it is firmly established that multiple KCOT are linked to Gorlin syndrome, i.e. mutation of PTCH gene, a few case reports of multiple KCOT without other manifestations of Gorlin syndrome were also reported in literature [12, 13]. Raghavendra et al. explained this occurrence by partial expression of PTCH gene [14]. This may also explain our case in which no skin lesions were observed and jaw cysts occurred in late adulthood.

In our case, based on clinical and radiologic findings, surgical excision was planned, however ideally such patients should first be biopsied to establish a diagnosis before embarking on definitive surgical management as complete removal of all cysts is key to prevent recurrence. Woolgar et al. compared the histologic features of KCOTs associated with Gorlin syndrome with those of solitary KCOTs. Syndromic KCOTs were found to be more significantly associated with satellite cysts, solid islands of epithelial proliferations, odontogenic rests within the capsule and in the number of mitotic figures in the lining epithelium [15]. Several authors have reported a higher recurrence rate of KCOTs particularly those associated with Gorlin syndrome. There are many reasons of this occurrence; for instance, by incomplete removal of the original cyst lining, by retention of daughter cysts, from epithelial islands in the wall of original cysts and by the development of new KCOTs from epithelial buds of the basal layer of oral epithelium [16]. This reinforces the importance of biopsy and proper surgical management.

Multidisciplinary clinical approach is needed to manage patients with Gorlin syndrome with the involvement of geneticists and maxillofacial surgeons. Moreover, dermatologists may also be involved to ensure early detection and treatment of basal cell carcinomas. KCOTs are hallmark of this disease and should always lead to its suspicion even in the absence of other clinical manifestations.

The two key features of Gorlin syndrome are basal cell carcinoma and KCOTs, both of which usually start to occur during 1st and 2nd decade of life. On the contrary, in our case, there was no evidence of basal cell carcinoma and there was even late occurrence of KCOTs. This may be explained by both genetic and environmental factors. Yaser et al. described nine genetic variants which modify the age of onset of basal cell carcinoma in Gorlin syndrome [17]. In addition, environmental factors like sunlight exposure and amount of skin pigmentation may also modify the age of presentation. Therefore a diagnosis of Gorlin syndrome should always be suspected in any patient presenting with KCOTs especially if multiple or recurring even if at a late age of onset. As in cases of Gorlin syndrome a more vigilant clinical approach would be needed including assessment of any developing skin lesions, aggressive surgical approach for the management of KCOTs, more closer clinical follow-up to deal with any recurring KCOTs and genetic counseling.

## Conclusions

Patients presenting with multiple KCOTs should always be screened for the presence of Gorlin syndrome, even in the absence of skin manifestations and late occurrence of cysts. As these patients are at increased risk of developing more KCOTs in their life time, therefore close clinical follow up is needed for their early detection. Additionally, genetic counseling with or without genetic testing should be considered for family members. We suggest a stepwise approach for the management of KCOTs which includes a preoperative biopsy to establish a definitive diagnosis, complete removal of all KCOTs to prevent recurrence and close clinical follow up. Moreover, a high index of suspicion of underlying Gorlin is necessary especially when there are multiple KCOTs as clinical features are markedly variable.

## Consent

Written informed consent was obtained from the patient for publication of this Case report and accompanying images. Ethics committee of Liaquat National hospital approved the study.

## Abbreviations

OKCs: odontogenic keratocysts
KCOT: keratocystic odontogenic tumors
